# Identifying and Validating Potential Biomarkers of Early Stage Lung Adenocarcinoma Diagnosis and Prognosis

**DOI:** 10.3389/fonc.2021.644426

**Published:** 2021-04-16

**Authors:** Yingji Chen, Longyu Jin, Zhibin Jiang, Suo Liu, Wei Feng

**Affiliations:** Department of Cardiothoracic Surgery, Third Xiangya Hospital of Central South University, Changsha, China

**Keywords:** early stage, lung adenocarcinoma, bioinformatics, biomarker, prognosis

## Abstract

**Background:**

Lung adenocarcinoma (LUAD) is the most common pathological type of lung cancer. At present, most patients with LUAD are diagnosed at an advanced stage, and the prognosis of advanced LUAD is poor. Hence, we aimed to identify novel biomarkers for the diagnosis and treatment of early stage LUAD and to explore their predictive value.

**Methods:**

The microarray datasets GSE63459, GSE27262, and GSE33532 were searched, and the differentially expressed genes (DEGs) were obtained using GEO2R. The DEGs were subjected to gene ontology (GO) and pathway enrichment analyses using METASCAPE. A protein–protein interaction (PPI) network was plotted with STRING and visualized by Cytoscape. Module analysis of the PPI network was performed using MCODE. Overall survival (OS) analysis and analysis of the mRNA expression levels of genes identified by MCODE were performed with UALCAN. Western blot analysis of hub genes in LUAD patients, MTS assays, and clonogenic assays were performed to test the effects of the hub genes on cell proliferation *in vitro*.

**Results:**

A total of 341 DEGs were obtained, which were mainly enriched in terms related to blood vessel development, growth factor binding, and extracellular matrix organization. A PPI network consisting of 300 nodes and 1140 edges was constructed, and a significant module including 15 genes was identified. Elevated expression of ASPM, CCNB2, CDCA5, PRC1, KIAA0101, and UBE2T was associated with poor OS in LUAD patients. In the protein level, the hub gene was overexpressed in LUAD patients. *In vitro* experiments showed that knockdown of the hub genes in the LUAD cell lines could promote cell proliferation.

**Conclusions:**

DEGs are potential biomarkers for early stage lung adenocarcinoma and could have utility for the diagnosis and predicting treatment efficacy.

## Introduction

Lung cancer is one of the most common malignancies and is the leading cause of cancer mortality (1). Non-small cell lung cancer (NSCLC) accounts for 80% of lung cancer cases (2), and adenocarcinoma is the most prevalent form of NSCLC, causing almost 40% of all lung cancers (3). Despite advances in the treatment of LUAD, such as targeted and immune therapy, the long-term survival of patients with LUAD is still poor (4, 5). Diagnosis at an advanced stage is one of the main reasons for the poor prognosis of LUAD patients. Hence, it is necessary to identify biomarkers that can be detected at the early stage of the disease and can serve as potential treatment targets.

Currently, high-throughput bioinformatics technologies such as microarrays are widely used to screen for differentially expressed genes (DEGs) (6). In the present study, we used well-established bioinformatics tools to screen potential biomarkers for the early diagnosis of LUAD. The Gene Expression Omnibus (GEO) database, an open-access database, was used to select appropriate mRNA profiles. The online analysis tools assisted in analyzing DEGs between tumor and normal tissues. We downloaded three microarray datasets, GSE63459, GSE27262, and GSE33532 from GEO. DEGs were obtained using GEO2R. Functional and pathway enrichment analysis was performed for DEGs using the METASCAPE online tool. A protein–protein interaction (PPI) network was established by using STRING, and it was visualized with Cytoscape. Module analysis of the PPI network was performed by using MCODE. Subsequently, overall survival (OS) analysis and mRNA expression of genes from MCODE were performed with UALCAN. Finally, several of the DEGs were tested *in vitro* to verify their functions.

## Materials and Methods

### Tissue Collection and Ethic Statement

Twelve primary LUAD patients undergoing surgical resection were collected at the Third Xiangya Hospital of Central South University (Changsha, China) from June 2019 to December 2019. None of the patients received chemotherapy or radiotherapy before surgery. Appropriate ethical approval was obtained from the Third Xiangya Hospital Ethics Committee, and written informed consent was obtained from all patients. Tissue specimens were snap frozen and kept in liquid nitrogen until further use.

### Data Acquisition

GEO is a public functional genomics data repository of high-throughput gene expression data, chips, and microarrays. Three gene expression datasets were downloaded from GEO. The GSE63459 dataset (GPL6883 Illumina HumanRef-8 v3.0 expression beadchip) contains 33 stage I LUAD tissues and 32 adjacent non-tumor tissues. The GSE27262 dataset (GPL570 HG-U133_Plus_2 Affymetrix Human Genome U133 Plus 2.0 Array) contains tumor and adjacent normal tissue pairs from 25 stage I LUAD patients. The GSE33532 dataset (GPL570 HG-U133_Plus_2 Affymetrix Human Genome U133 Plus 2.0 Array) contains 40 stage I and II LUAD tumor samples and 10 normal samples.

### Identification of DEGs

The DEGs between tumor and normal tissue were detected by GEO2R. GEO2R is a powerful interactive online tool that allows users to screen differentially expressed mRNAs between two or more groups in GEO series. A P value <0.05 and |logFC|>1 were used as selection criteria.

### Pathway and Functional Enrichment Analysis

Gene Ontology and Kyoto Encyclopedia of Genes and Genomes (KEGG) pathway enrichment analyses were performed for the identified DEGs using METASCAPE (https://metascape.org/), a gene annotation and analysis resource. A P value <0.05 was used to distinguish significantly enriched genes.

### Integration of the PPI Network

STRING is an online tool designed to evaluate PPI (protein–protein interaction networks) information. To detect the potential relationships among the DEGs, the STRING database was used to construct a PPI network. Cytoscape, a free visualization software, was applied to visualize the PPI network. In addition, the Molecular Complex Detection (MCODE) app in Cytoscape was used to check the modules of the PPI network (degree cutoff = 2, max. depth = 100, K-core = 2, and node score cutoff = 0.2).

### Survival Analysis and Expression Level of the Hub Genes

The survival analysis and the evaluation of the mRNA expression levels at different tumor stages of the hub genes were performed using UALCAN (http://ualcan.path.uab.edu), a user-friendly, interactive web resource for analyzing cancer transcriptome data from The Cancer Genome Atlas (TCGA). A P value <0.01 was used to select the hub genes for the survival analysis.

### Cell Culture and siRNA-Mediated Knockdown

The A549 and H460 LUAD cell lines obtained from ATCC (Gaithersburg, MD, USA) were cultured in RPMI-1640 containing 10% fetal bovine serum in a humidified 5% CO_2_ incubator at 37°C. Small interfering RNAs (siRNAs) against ASPM, CCNB2, CDCA5, KIAA0101, PRC1, UBE2T, and negative control siRNAs (NC-siRNA), which were obtained from GeneChem (Shanghai, China), were transfected into cell lines using Lipofectamine 2000 transfection reagent (Invitrogen, Shanghai, China) according to the manufacturer’s instructions.

### Western Blot Analysis

Cells were harvested and lysed in RIPA buffer (CWbio, Beijing, China) containing 0.1 mg/ml PMSF (Keygen, Nanjing, China) and protease inhibitors (Roche, Mannheim, Germany). The western blot procedure was the same as that described in our previous research (7). Antibodies against ASPM, CCNB2, CDCA5, PRC1, UBE2T, goat anti-rabbit IgG-HRP antibodies, and goat anti-mouse IgG-HRP were purchased from Proteintech (Wuhan, China). The antibody KIAA0101 was purchased from Cell Signaling Technology (Danvers, USA).

### Cell Proliferation Assay

Cell proliferation was assessed using MTS and clonogenic assays. For the MTS assay, transfected A549 and H460 cells were seeded (2 × 10^3^ cells/well in 200 μl) into 96-well plates and divided into the si-control group (four wells) and the si-hub gene groups (four wells). After incubation for 0, 24, 48, or 72 h, MTS reagent (Promega, Madison, USA) was added to assess cell viability according to the manufacturer’s instructions. For the clonogenic assay, transfected A549 and H460 cell lines were seeded (1 × 10^3^ cells/well) into six-well plates and divided into a si-control and si-hub gene groups. After 1–2 weeks of culture, the colonies were stained with crystal violet and analyzed using Image-Pro Plus 7.0 software.

### Statistical Analysis

All *in vitro* experiments were repeated three or more times. All values were mean ± standard deviation. Statistical significance was measured with PRISM 8. P <0.05 was considered statistically significant.

## Results

### Identification of DEGs

**Figure 1:**
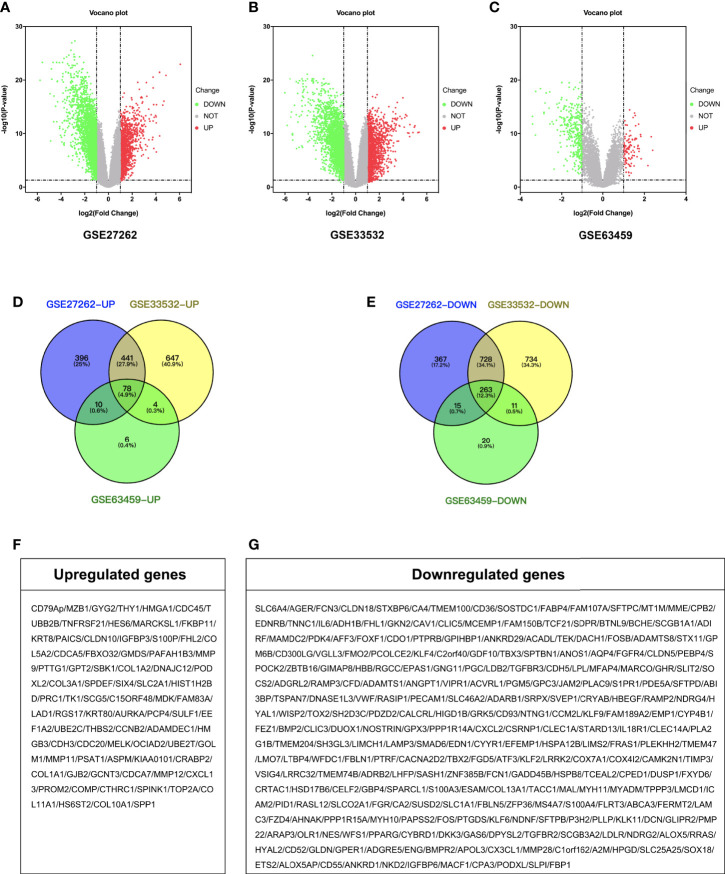
Identification of DEGs. Volcano plot of the distribution of all differentially expressed genes including GSE27262, GSE33532, and GSE63459. **(A–C)** Venn diagram of **(D)** up-regulated and **(E)** down-regulated DEGs were selected with P < 0.05 and fold change >2 among the mRNA expression profiling sets GSE27262, GSE33532, and GSE63459. **(F, G)** Gene symbol of up-regulated and down-regulated DEGs.

A total of 2,298 DEGs were successfully identified in early stage LUAD following GSE27262 dataset analysis, including 925 up-regulated and 1,373 down-regulated genes (**Figure 1A**). A total of 2,906 DEGs were extracted from the GSE33532 dataset, including 1,170 up-regulated and 1,736 down-regulated genes (**Figure 1B**). In the GSE63459 dataset, 407 DEGs involving 98 up-regulated and 309 down-regulated genes were found (**Figure 1C**). Then, we used Venn diagram software to identify the common DEGs in the three datasets. A total of 341 common DEGs in early stage LUAD tissues were detected, including 78 up-regulated genes and 263 down-regulated genes (**Figures 1D–G**).

### GO and KEGG Enrichment Analyses of the DEGs

**Figure 2:**
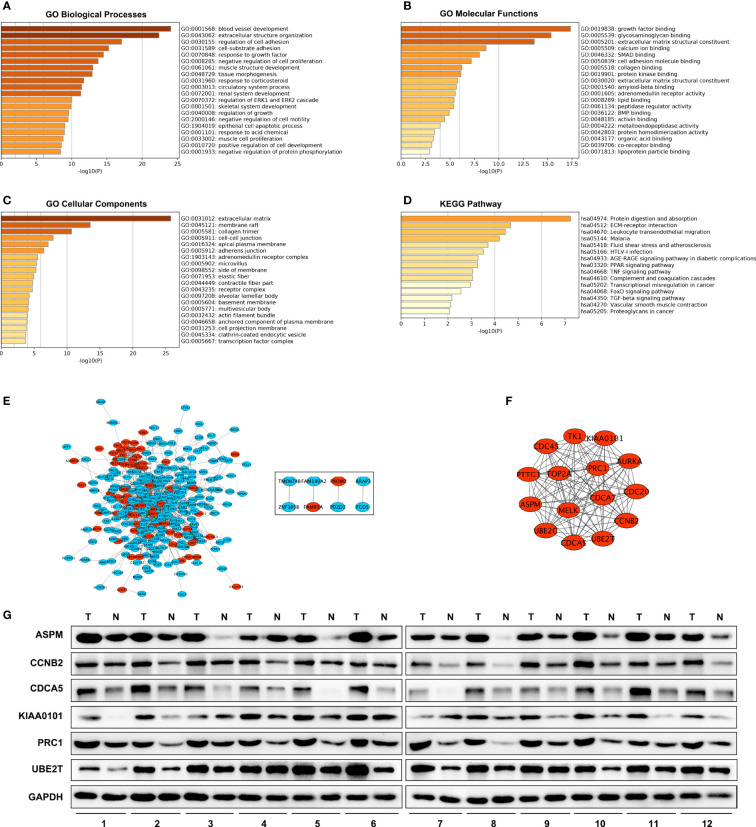
GO, KEGG and PPI network. GO enrichment analysis with all DEGs. **(D)** KEGG Pathway analysis with all DEGs. **(A–C)** PPI network of DEGs. **(E)** A significant module selected from protein–protein interaction network. Red nodes are up-regulated genes, blue nodes are down-regulated genes. **(F)** Western blot analysis of hub genes protein level expression in tumor tissues (T) and adjacent non-malignant lung tissues (N) in LUAD patients **(G)**.

BP (biological processes) analysis demonstrated that a total of 341 DEGs were dramatically enriched in blood vessel development, extracellular structure organization, and regulation of cell adhesion (**Figure 2A**). MFs (molecular functions) showed that the overlapping DEGs were significantly enriched in growth factor binding, glycosaminoglycan binding and extracellular matrix structural constituents (**Figure 2B**). CCs (cellular components) showed that the overlapping DEGs were significantly concentrated in the extracellular matrix, membrane raft, and collagen trimer (**Figure 2C**). In addition, KEGG analysis revealed that all DEGs were mainly enriched in protein digestion and absorption, ECM–receptor interaction and leukocyte transendothelial migration (**Figure 2D**).

### PPI Network Construction and Analysis of Modules

A total of 300 DEGs were imported into the DEG PPI network complex, which included 300 nodes and 1,140 edges, containing 232 down-regulated genes and 68 up-regulated genes (**Figure 2E**). Then, we applied MCODE for further analysis, and the results showed 15 central nodes, which were all up-regulated genes, among the 300 nodes (**Figure 2F**).

### Analysis of Hub Genes by UALCAN

**Figure 3:**
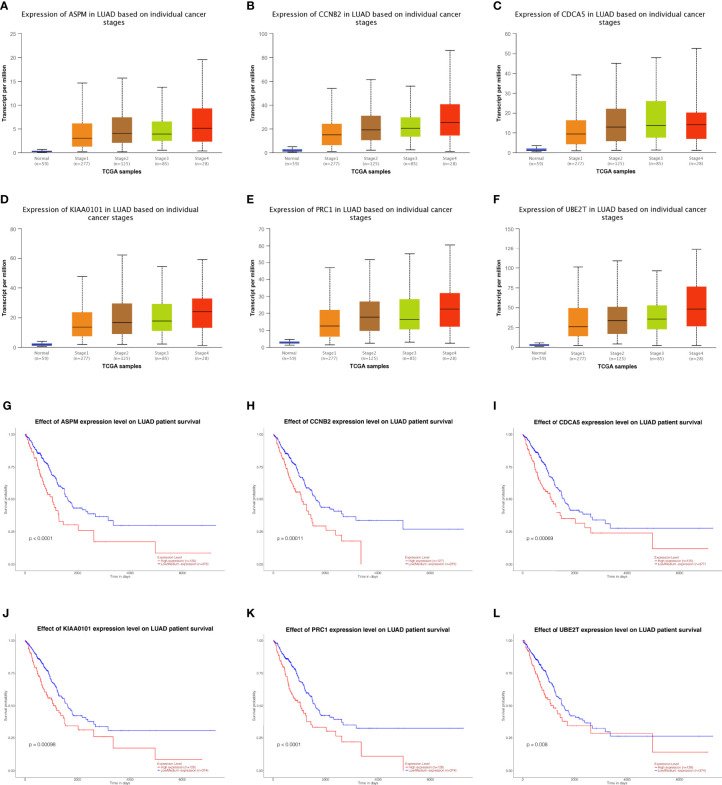
Analysis of hub genes. Expression of genes in different stages including ASPM, CCBN2, CDCA5, KIAA0101, PRC1, and UBE2T in lung adenocarcinoma patients. **(A–F)** Prognostic estimation of genes including ASPM, CCBN2, CDCA5, KIAA0101, PRC1, and UBE2T in lung adenocarcinoma patients **(G–L)**.

Fifteen genes in the PPI network were evaluated for their prognostic value by UALCAN. Only six genes, including ASPM, CCNB2, CDCA5, KIAA0101, PRC1, and UBE2T, exhibited potential in the prediction of survival based on their expression level (**Figures 3G–L**). In addition, the mRNA expression levels of the six genes were dramatically increased in early stage tumor tissues relative to normal tissues (**Figures 3A–F**).

### The Hub Genes Were Up-Regulated in LUAD Tissues Compared With Normal Lung Tissues

We investigated the expression of ASPM, CCNB2, CDCA5, KIAA0101, PRC1, and UBE2T in 12 LUAD tumor tissues and their adjacent non-malignant lung tissues. The results also showed these hub genes were overexpressed in LUAD patient samples (**Figure 2G**). These results indicated that hub genes are overexpressed in LUAD cells and might promote tumor genesis.

### Validation of the Hub Genes *In Vitro*

**Figure 4:**
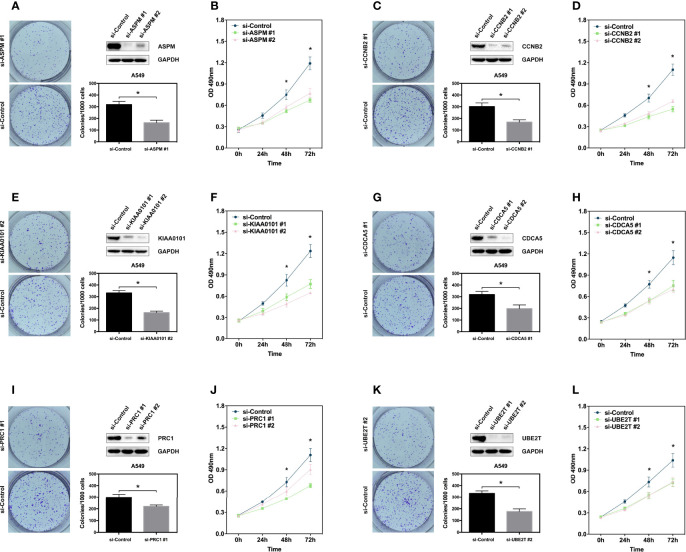
Hub genes promote proliferation of lung adenocarcinoma cell. **(A, C, E, G, I, K)** Clone formation assay showing the proliferation ability of the A549 cells with genes knockdown including ASPM, CCBN2, CDCA5, KIAA0101, PRC1, and UBE2T. Western blot showing the expression of hub genes in A549 cells. **(B, D, F, H, J, L)** MTS assay showing the proliferation ability of the A549 cells with genes knockdown including ASPM, CCBN2, CDCA5, KIAA0101, PRC1, and UBE2T. *P < 0.05.

To further validate the relationship between the hub genes and LUAD, we generated transient hub gene knockdown A549 LUAD cells. We found that knockdown of these genes significantly inhibited their clonogenic ability and proliferation (**Figures 4A–L**). The same results were observed in H460 cell lines (**Supplementary Figure 1**). These results demonstrate that the six hub genes are potential biomarkers for early stage LUAD diagnosis and prognosis.

## Discussion

Regardless of the comprehensive treatments available in the clinic, LUAD has a high mortality. The main reason is the lack of sufficient screening methods for early stage LUAD. To improve the prognosis of the patients, we attempted to identify biomarkers of LUAD that can be used for screening. In this study, bioinformatics analysis was performed to identify the candidate key genes correlated with early stage LUAD.

By comparing the three DEG profiles of early stage LUAD from the GEO datasets, 78 up-regulated and 263 down-regulated DEGs were selected. Then, a total of 341 DEGs were analyzed for the functions using GO terms and KEGG pathways. The BPs were abundantly enriched in blood vessel development, extracellular structure organization, and regulation of cell adhesion. The MFs were enriched in growth factor binding, glycosaminoglycan binding, and extracellular matrix structural constituent. The CCs were enriched in extracellular matrix, membrane raft, and collagen trimer. For pathway analysis, the DEGs were particularly enriched in protein digestion absorption, ECM–receptor interaction and leukocyte transendothelial migration. This GO term and KEGG pathway analysis revealed that the DEGs were significantly associated with extracellular matrix-related functions. A previous study showed that the extracellular matrix has crucial roles in tumor metastasis (8).

Next, a DEG PPI network complex of 300 nodes and 1,140 edges was constructed *via* the STRING online database and Cytoscape software. Then, 15 vital up-regulated genes were extracted from the PPI network complex by Cytotype MCODE analysis. Furthermore, through Kaplan–Meier plotter analysis, we found that the expression levels of seven of the 15 genes had a correlation with a significantly worse survival. In addition, the mRNA expression levels of six genes, ASPM, CCNB2, CDCA5, KIAA0101, PRC1, and UBE2T were overexpressed in early stage LUAD relative to normal tissues.

ASPM (abnormal spindle microtubule assembly) is connected to the development of diverse tumors. For example, ASPM expression is incrementally up-regulated in primary and metastatic prostate cancer (9). In addition, ASPM is associated with cell cycle progression in pancreatic ductal adenocarcinoma (10) and cell proliferation in lung squamous cell carcinoma (11). However, the role of ASPM in LUAD has rarely been reported.

CCNB2 (Cyclin B2), a member of the cyclin family of proteins, plays a key role in the progression of the G2/M transition (12–14). Recently, studies have suggested that CCNB2 expression is increased in a variety of human cancers. CCNB2 functions as an oncogene and could serve as a potential biomarker of an unfavorable prognosis over short-term follow-up in breast cancer (15). In hepatocellular carcinoma, CCNB2 may serve as a prognostic factor and participate in tumor development and progression by promoting cell proliferation and migration (16). Regarding lung cancer, Qian X et al. showed that CCNB2 overexpression is positively associated with the degree of differentiation, tumor size, lymph node metastasis, distant metastasis, and clinical stage in NSCLC (17).

CDCA5 (Cell Division Cycle Associated 5) is required for stable binding of cohesion to chromatid in the S and G2/M phases and is degraded through anaphase-promoting complex-dependent ubiquitination in the G0/G1 phase (18–21). CDCA5 expression is elevated and is associated with a poor prognosis in several human cancers, such as urothelial carcinoma and oral squamous cell carcinoma (22, 23). In addition, knockdown of CDCA5 could inhibit cancer cell growth by arresting the cell cycle in the G2/M phase and promoting apoptosis (23). In lung carcinoma, CDCA5 and its phosphorylation at Ser209 by ERK play an important role in lung cancer cell proliferation (24).

KIAA0101 (PCNA-associated factor) is a supplementary factor for DNA polymerase and is required for DNA replication or repair (25). KIAA0101 functions as an oncogene and is up-regulated in many cancers. W. Lv et al. found that inhibition of KIAA0101 could suppress cell proliferation and cell cycle progression in breast cancer (26). Similarly, M. Hosokawa et al. revealed that suppression of KIAA0101 caused a drastic attenuation of cell proliferation and a significant decrease in the DNA replication rate in pancreatic cancer (27). Consistent with our work, studies have shown that KIAA0101 could regulate the cell cycle of NSCLC and that high-level KIAA0101 expression could serve as an independent prognostic factor in NSCLC (28–30).

PRC1 (Protein Regulator of Cytokinesis-1) is characterized as a mitotic spindle-associated cyclin-dependent kinase (CDK) substrate (31). It has already been reported that PRC1 is overexpressed in different cancers, such as hepatocellular carcinoma and pancreatic cancer (32, 33). A mechanistic study showed that PRC1 could promote LUAD cell proliferation, invasion, and metastasis by limiting G2/M phase cell cycle arrest and apoptosis (34).

UBE2T (ubiquitin-conjugating enzyme E2T), a typical ubiquitin-conjugating enzyme, connects with a particular E3 ubiquitin ligase to degrade related substrates (35). Numerous studies have identified that its overexpression is involved in tumorigenesis in a number of different types of cancer (36). For example, UBE2T promotes breast cancer cell proliferation by inhibiting the expression of BRAC1 (37). The expression of UBE2T increases with the progression of multiple myeloma, especially in the early stage (38). UBE2T silencing inhibited non-small cell lung cancer cell proliferation and invasion by suppressing the Wnt/*β*-catenin signaling pathway (39).

To provide strong support for the results of our bioinformatics analyses, we carried out *in vitro* experiments. We verified that silencing the hub genes separately in LUAD cell lines could decrease cell viability, and we observed that these hub genes’ protein levels were up-regulated in LUAD patients. These results indicate that the six hub genes may play a vital role in LUAD. Nevertheless, the underlying molecular mechanisms of these hub genes in the development and progression of early stage LUAD remain to be further explored.

## Data Availability Statement

The original contributions presented in the study are included in the article/**Supplementary Material**. Further inquiries can be directed to the corresponding author.

## Author Contributions

(I) Conception and design: YC, LJ, WF. (II) Administrative support: WF. (III) Provision of study materials or patients: YC, LJ. (IV) Collection and assembly of data: ZJ, SL. (V) Data analysis and interpretation: YC, LJ (VI) Manuscript writing: All authors (VII) All authors contributed to the article and approved the submitted version.

## Funding

This study was supported by Hunan Science and Health Union foundation (No. 2018JJ6135 and No. 2018JJ6053).

## Conflict of Interest

The authors declare that the research was conducted in the absence of any commercial or financial relationships that could be construed as a potential conflict of interest.
