# Supplementary figures and images for: Identifying and Validating Potential Biomarkers of Early Stage Lung Adenocarcinoma Diagnosis and Prognosis
Source: Front Oncol. 2021 Apr 16;11:644426. doi: 10.3389/fonc.2021.644426 (PMC8085413; doi:10.3389/fonc.2021.644426)

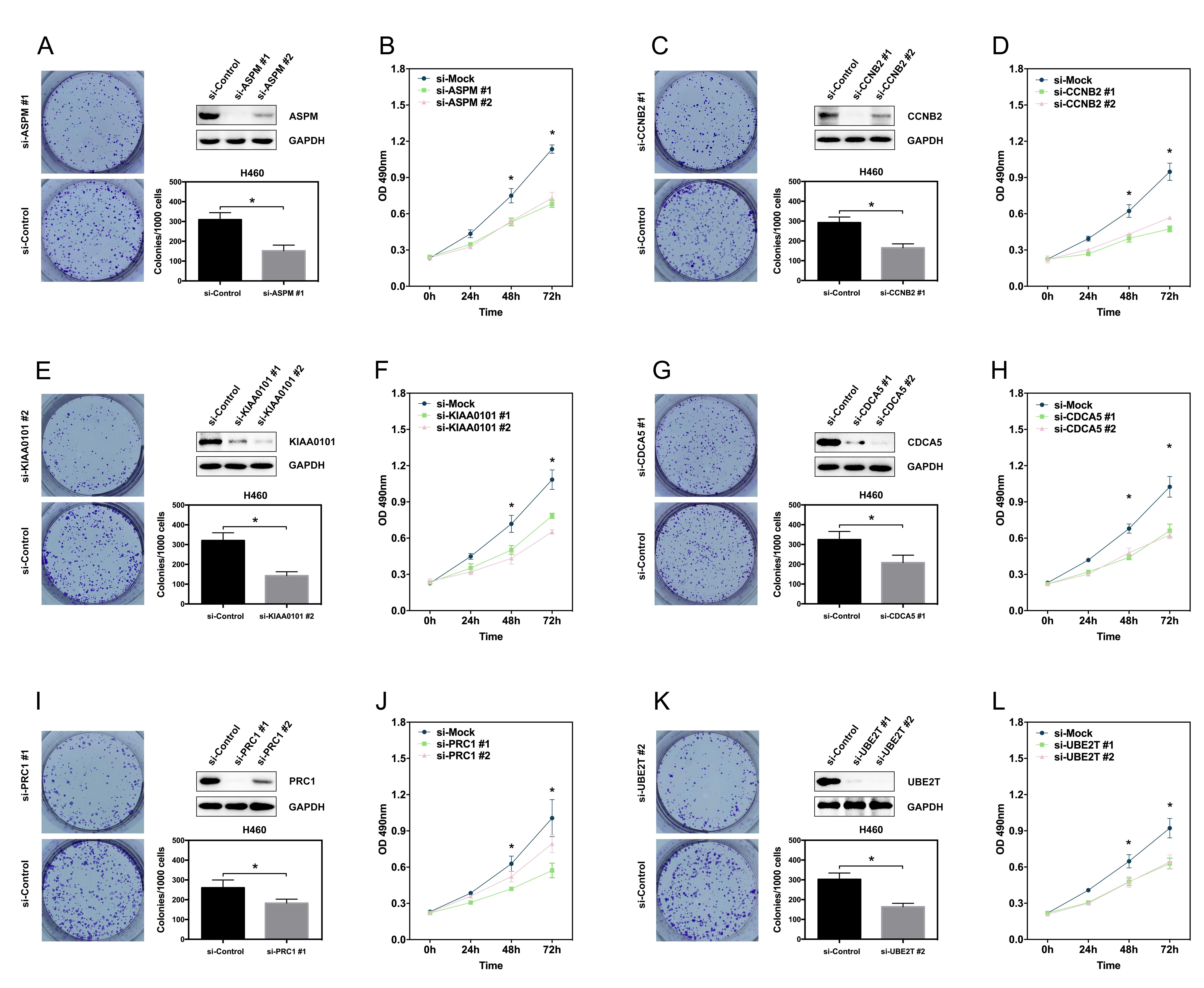

Supplement: Supplementary file 1 [file Image_1.jpeg]
